# An Unusual Case of Zero Percent Coagulopathic Factor in a Patient With Polymyalgia Rheumatica

**DOI:** 10.7759/cureus.33414

**Published:** 2023-01-05

**Authors:** Nathalie De Paz, Miguel A Belaunzaran, Ruben Cabrera, Tommy Macias, Sherard Lacaille, Carlos Guida

**Affiliations:** 1 Internal Medicine, Kendall Regional Medical Center, Miami, USA; 2 Medicine, Dr. Kiran C. Patel College of Allopathic Medicine, Davie, USA; 3 Critical Care, Kendall Regional Medical Center, Miami, USA; 4 Hematology and Oncology, Kendall Regional Medical Center, Miami, USA

**Keywords:** case report, factor viii inhibitors, factor viii deficiency, polymyalgia rheumatica, acquired hemophilia a

## Abstract

We present a case of a 74-year-old male with a past medical history of polymyalgia rheumatica that presented as a transfer for evaluation of hematomas of the scrotum, left groin, back, and bilateral thighs. Further questioning revealed hematuria and bleeding gums for the past month. The patient complained of left thigh pain without recent fever, chills, chest pain, or shortness of breath. A physical exam showed hematomas of the left groin, scrotum, bilateral thighs, and back with an ecchymotic appearance.

Initial pertinent laboratory workup showed decreased hemoglobin, leukocytosis, and elevated partial thromboplastin time (PTT). Therefore, a decision was made to obtain a CT angiogram of the abdomen and pelvis, which revealed retroperitoneal hematoma. Further diagnostic workup showed a coagulation factor VIII level of zero percent and mixing studies supporting the presence of an acquired factor VIII inhibitor. Therefore, the patient was treated with rituximab and recombinant factor VIIa, with an improvement of factor VIII levels to normal limits within a week.

## Introduction

Bleeding disorders related to coagulation factors can be due to decreased or absent production of the coagulation factor or the presence of an acquired factor inhibitor. These acquired inhibitors have been reported for all coagulation factors. For example, acquired hemophilia A is due to an acquired inhibitor against coagulation factor VIII. Disease incidence is estimated to be 1-2 cases per million per year. Approximately half of acquired factor VIII inhibitors cases have associated conditions such as malignancy and autoimmune diseases. This condition should be investigated in patients with bleeding diathesis and an isolated increment of the activated partial thromboplastin time (aPTT) [1].

Several methods have been developed to detect the presence of acquired inhibitors. Mixing studies indicate the presence of these acquired inhibitors when the aPTT does not correct after mixing the patient's plasma with normal plasma. Nijmegen-modified Bethesda assay is another method that can detect and quantify the auto-antibodies present. Antifactor VIII enzyme-linked immunosorbent assay can also detect acquired inhibitors, especially if lupus anticoagulant interferes with Bethesda assay or treatment with recombinant porcine factor VIII has been started. Treatment involves controlling bleeding diathesis and addressing the underlying acquired antibody inhibitors [2].

Available treatments for bleeding control include bypassing agents such as recombinant factor VII, recombinant porcine factor VIII, and prothrombin complex concentrate. In addition, immunosuppressive regimens are used to eliminate acquired inhibitors, including corticosteroids, cyclophosphamide, rituximab, and combinations of these. The median time to achieve remission is five weeks [2].

## Case presentation

We present a 74-year-old male with a past medical history of polymyalgia rheumatica, hypertension, Barrett esophagus, urinary tract infections, and hematuria. He presented to the ED as a transfer from another hospital. The patient transferred due to ecchymosis concerning hematomas, low hemoglobin, and no nearby urologist in the region. He additionally reported hematuria and bleeding gums after brushing his teeth for the past month. In the ED, the patient complained of left thigh pain only. He denied having a recent fever, chills, chest pain, or shortness of breath. However, on arrival at the ED, a physical exam of the patient revealed hematomas of the left groin, scrotum, bilateral thighs, and back with an ecchymotic appearance (Table 1). 

**Table 1:** Laboratory values. PTT: Partial thromboplastin time; INR: International normalized ratio; CRP: C-reactive protein.

Laboratory values with reference ranges		
Hemoglobin	7.5 g/dL	13.7-17.5 g/dL
WBCs	11.3 x 10^3^	(4.0-10.5) x 10^3^
Fibrinogen	693 mg/dL	207-473 mg/dL
PTT	72 seconds	25-38 seconds
INR	1.1	0.0-1.1
CRP	8.1 mg/dL	0.0-1.0 mg/dL
Coagulation factor VIII	0%	50-150%
Von Willebrand factor antigen	231.8%	50-168%
Circulating anticoagulant aPTT (pre-mixing)	97 seconds	25.1-36.5 seconds
PTT (80:20 patient to normal plasma mixing ratio)	84.2 seconds	25.1-36.5 seconds
PTT (50:50 patient to normal plasma mixing ratio)	69.3 seconds	25.1-36.5 seconds
PTT (20:80 patient to normal plasma mixing ratio)	52.1 seconds	25.1-36.5 seconds
Porcine factor VIII titer	26.9 Bethesda units	0.0-0.8 Bethesda units
Ristocetin factor assay	50-200%	50-200%
Carcinoembryonic antigen	6.5 ng/mL	0.0-5.0 ng/mL
Cancer antigen 19-9	<15 units/mL	0.0-30.9 units/mL

**Figure 1:**
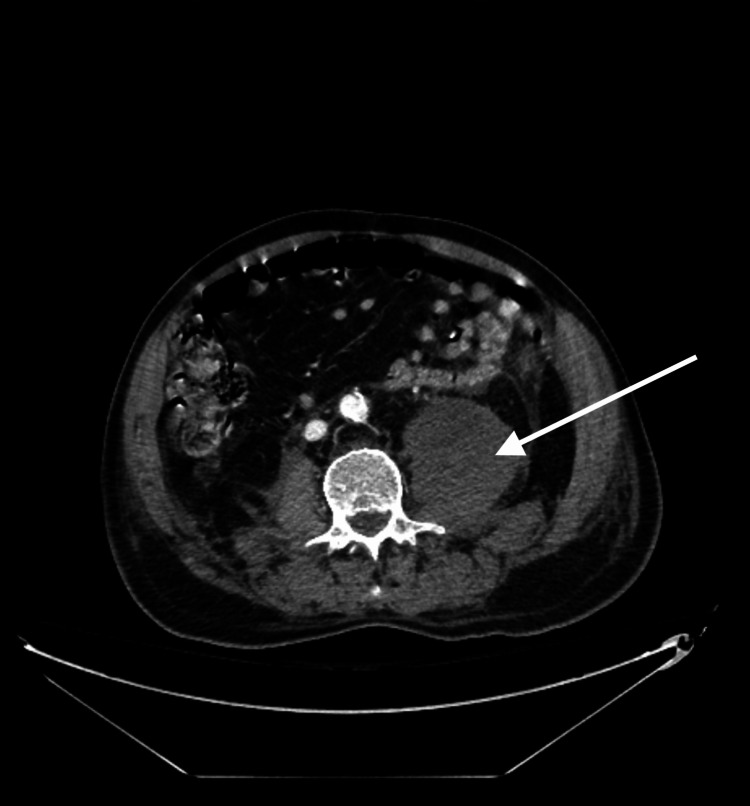
Cross-sectional view of retroperitoneal hematoma.

**Figure 2:**
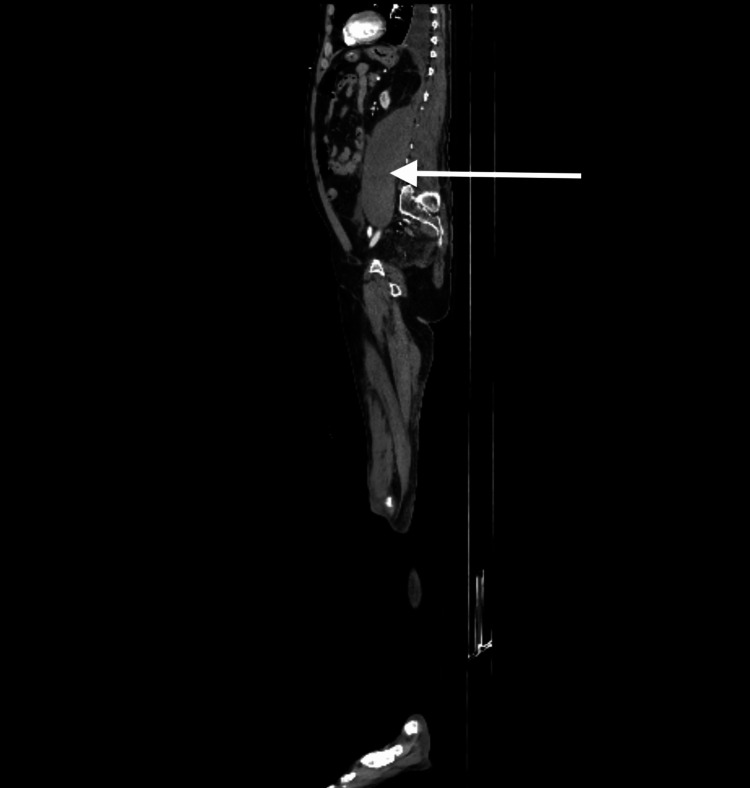
Sagittal view of retroperitoneal hematoma.

**Figure 3:**
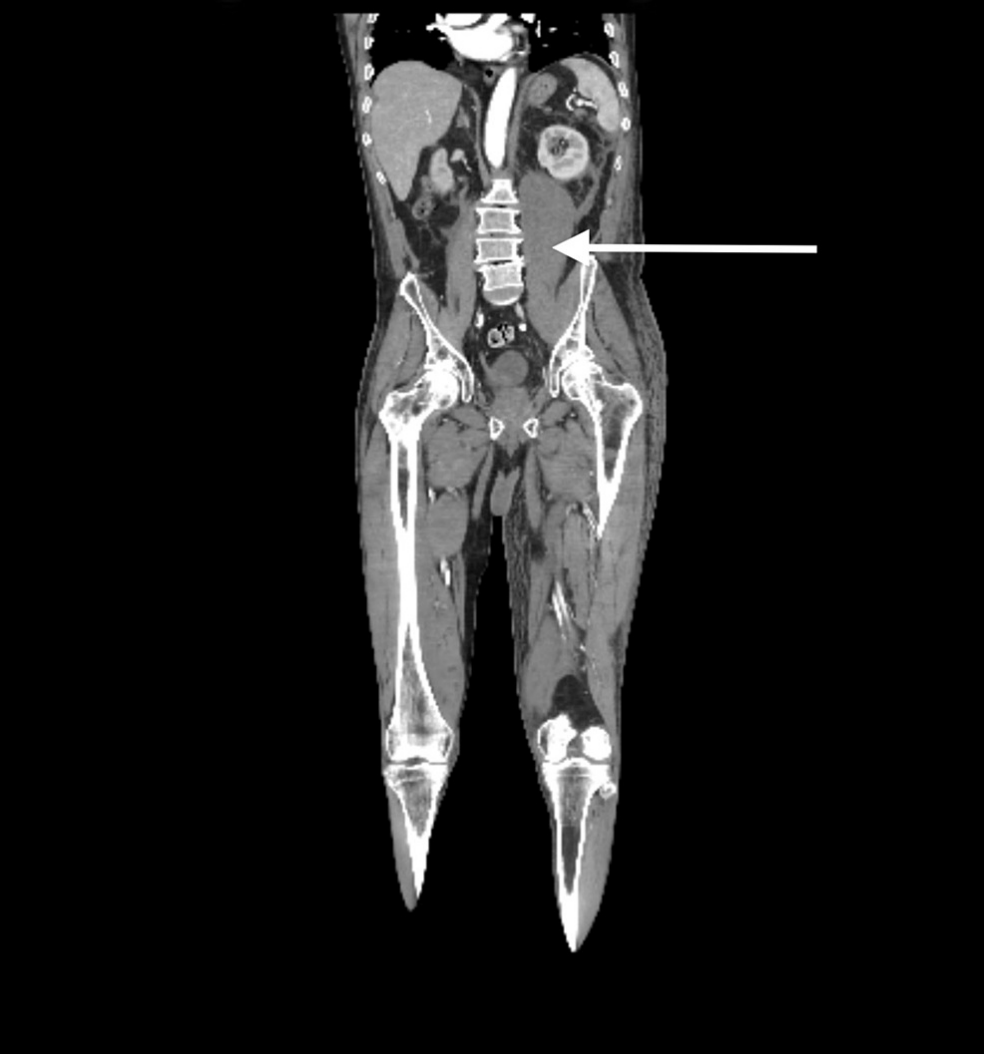
Coronal view of retroperitoneal hematoma.

The patient tested negative for rheumatoid factor and antinuclear antibody. In the subsequent days, the patient's hemoglobin decreased below seven g/dL, and a blood transfusion was administered. Further workup of the patient revealed masses in the bladder and esophagus. Biopsies of these masses were negative for malignancy. Due to concern of active bleeding and extravasation, a CT angiogram of the abdomen and pelvis revealed a left psoas retroperitoneal hematoma with no active extravasation (Figures 1-3). The patient received recombinant factor VIIa and rituximab therapy. Coagulation factor VIII levels improved to 64% within one week of treatment. 

## Discussion

Here we report a case of a 74-year-old man with a history of polymyalgia rheumatica that developed acquired factor VIII deficiency due to a factor VIII inhibitor. The patient developed bleeding diathesis, which included hematuria and a retroperitoneal hematoma. Factor VIII level obtained initially was zero percent. The aPTT levels did not correct with mixing studies. The patient was treated with recombinant factor VIIa to address the bleeding events and rituximab to eradicate the underlying factor VIII inhibitor. We suggest that polymyalgia rheumatica could be involved in the development of this inhibitor. Gallant M et al. reported in 2008 a patient with polymyalgia rheumatica/arteritis temporalis that developed an acquired factor VIII inhibitor. They treated the patient with oral doses of 64 mg methylprednisolone and cyclophosphamide 100 mg per day. The treatment improved symptoms that disappeared within two days and undetectable factor VIII inhibitor levels within four weeks. They proposed adding polymyalgia rheumatica/arteritis temporalis to the list of autoimmune causes of acquired hemophilia [3].

Bruegel M et al. report a patient with polymyalgia rheumatica treated with prednisone and leflunomide that developed hematomas. The patient was diagnosed with acquired factor VIII deficiency. Treatment with prothrombin complex concentrate, oral prednisone, and cyclophosphamide resulted in minimal improvement in factor VIII levels. However, treatment with rituximab achieved full remission of factor VIII activity and undetectable inhibitor level [4]. In our case, treatment with cyclophosphamide was not pursued due to its side effect profile. In addition, cyclophosphamide can cause bladder toxicity with resultant conditions such as hemorrhagic cystitis; this was an unfavorable choice given our patient's history of hematuria and bladder mass [5]. Several autoimmune conditions are associated with acquired factor VIII deficiency, including systemic lupus erythematosus, rheumatoid arthritis, and Sjogren's syndrome [3]. We add to the current literature another case of acquired hemophilia associated with polymyalgia rheumatica.

## Conclusions

Factor VIII deficiency secondary to an acquired coagulation factor inhibitor is a rare disease with known associations with malignancy and autoimmune diseases. Therefore, this condition should be suspected in patients with an isolated elevated aPTT, with follow-up diagnostic methods including mixing studies and Nijmegen-modified Bethesda assay. In addition, polymyalgia rheumatica is an autoimmune condition previously reported as one of the causes of acquired hemophilia A. We present a case of a 74-year-old male with known polymyalgia rheumatica that presented with a bleeding diathesis accompanied by an isolated aPTT elevation and an undetectable level of coagulation factor VIII.
